# Impact of Perfluorinated Organic Acids on Bacterial Ice Nucleators

**DOI:** 10.1021/acs.jpcb.5c07036

**Published:** 2026-02-10

**Authors:** Galit Renzer, Rosemary J. Eufemio, Mischa Bonn, Konrad Meister

**Affiliations:** † Department for Molecular Spectroscopy, Max Planck Institute for Polymer Research, 55128 Mainz, Germany; ‡ Department of Chemistry and Biochemistry, 1791 Boise State University, Boise, Idaho 83725, United States

## Abstract

Perfluorinated acids such as perfluorooctanoic acid (PFOA) and perfluorooctanesulfonic acid (PFOS) constitute major environmental pollutants with largely unknown effects on biological ice nucleators (INs), which are crucial for freezing-associated processes in nature. One of the most efficient and abundant INs are bacterial ice-nucleating proteins (INpros). Their record-breaking freezing efficiency relies on assembling large functional aggregates, which, while highly active, show high sensitivity to environmental conditions. This study demonstrates that PFOS and PFOA destroy INpro aggregates, significantly reducing the bacterial ice nucleation efficiency near 0 °C. Exposure to perfluorinated compounds at concentrations of 10 mg/L is sufficient to alter the bacterial IN activity and make them highly unstable toward repetitive freezing. We propose that the adverse effects are based on a coupled mechanism that interrupts attractive electrostatic interactions relevant for INpro multimerization by proton-mediated charge screening, direct electrostatic interference by deprotonated PFOS and PFOA headgroups, and perturbation of the assembly-facilitating bacterial membrane. Our findings contribute to evaluating the ongoing environmental risks of perfluorinated compounds and their effects on related ecological and atmospheric processes.

## Graphical abstract

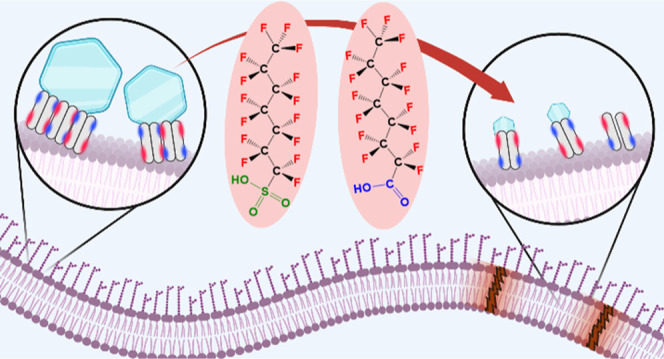

## Introduction

Biological ice nucleators (INs) influence freezing processes across atmospheric and terrestrial environments, yet little is known about the influence of anthropogenic contaminants on their activity. Among the most efficient biological INs are ice-nucleating bacteria, such as *Pseudomonas syringae*,^1,2^ which catalyze ice formation at temperatures close to 0 °C through specialized ice-nucleating proteins (INpros). These bacteria are encountered in diverse environments including plant surfaces, atmospheric aerosols, clouds, rain, snow, and hail,^2–11^ indicating their role in cloud glaciation and influencing precipitation patterns.^3–8,12–14^ In parallel, per- and polyfluorinated alkyl substances (PFAS), which have emerged as persistent anthropogenic pollutants often referred to as “forever chemicals”, now contaminate all major environmental compartments globally,^15,16^ accumulating in the same ecological spheres where these bacteria thrive. Given that both PFAS and bacterial INs are globally distributed through similar transportation mechanisms^3,15–17^ and concentrate at air–water interfaces such as cloud droplets,^18,19^ their interactions pose an unrecognized threat to atmospheric freezing processes and climate-relevant biological functions.

Ice nucleation governs key natural processes including cloud microphysics, weather patterns, and the survival of cold-adapted organisms. At high subzero temperatures, freezing is primarily mediated by heterogeneous ice nucleators,^20–22^ which are indispensable for maintaining ecological and hydrological cycles and for regulating earth’s climate. Bacterial INpros are among the most efficient and abundant INs and achieve their exceptional efficiency through the assembly of large functional aggregates on the bacterial outer membrane,^23,24^ as illustrated in Figure 1A. Freezing assays reveal that bacterial INs are active across a broad spectrum of temperatures ranging from −2 to −12 °C. Based on their freezing efficiency, bacterial INs are commonly classified into classes A to C, with characteristic freezing temperatures of −4.4 °C or warmer (class A), −4.6 to −5.7 °C (class B), and −7.6 °C and colder (class C),^25^ noting that exact temperature boundaries can vary slightly with assay conditions. These differences in activity arise from INpro aggregates of different sizes, with class A INs comprising the largest and most active assemblies.^23,26,27^ Recent research suggests a hierarchical supramolecular mechanism for INpro aggregation, where dimers initially form through tyrosine interactions, followed by their assembly through electrostatic interactions.^27^ This assembly leads to the functional multimerization of INpros and requires an intact bacterial cell membrane.^28,29^ Several studies have demonstrated that bacterial INs are highly sensitive to changing environmental conditions (e.g., pH, salts, temperature, cosolutes), with larger aggregates—responsible for their high freezing efficiency—being particularly affected.^30–32^

**Figure 1:**
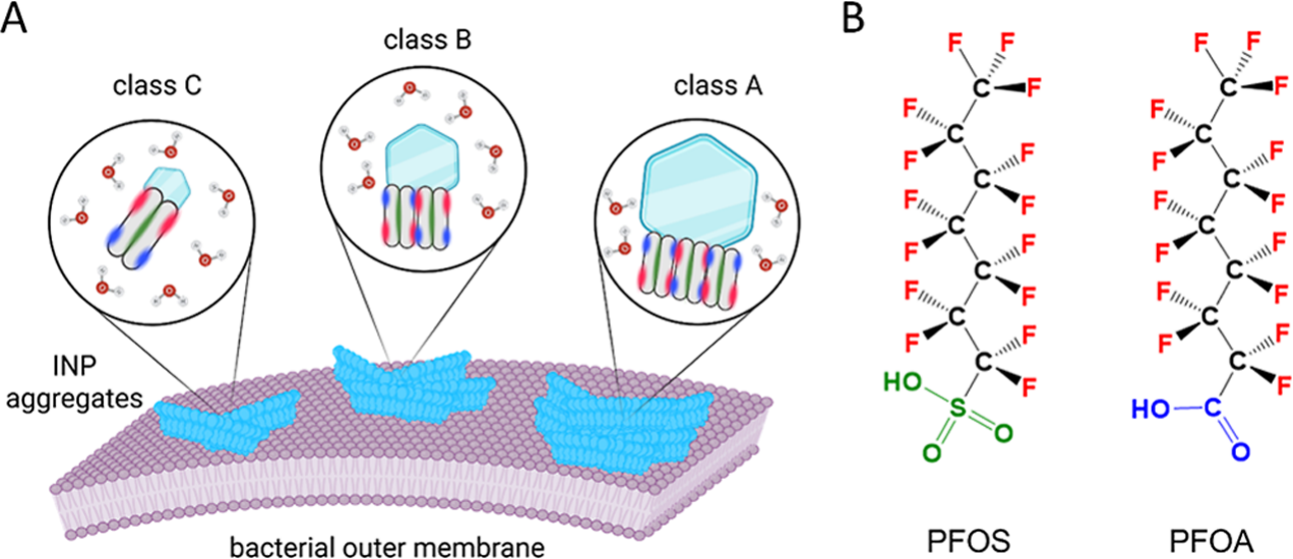
(A) Schematic representation of bacterial ice-nucleating proteins (INpros) located in the bacterial outer membrane of *P. syringae*, showing the proposed assembly mechanism for INpro multimerization: Dimer formation occurs through tyrosine interactions (green), whereas larger aggregates form through electrostatic attraction between positively (blue) and negatively (red) charged amino acid side chains. The freezing efficiency of INpro aggregates increases with aggregate size, with class A comprising the largest and most efficient aggregates and class C comprising the smallest, less active aggregates. (B) Chemical structures of the investigated perfluorinated organic acids, perfluorooctanesulfonic acid (PFOS) and perfluorooctanoic acid (PFOA).

Over recent decades, PFAS have emerged as significant and ubiquitous environmental pollutants, with bioaccumulating, persistent, and toxic properties.^33,34^ Among PFAS, perfluorooctanoic acid (PFOA) and perfluorooctanesulfonic acid (PFOS) (Figure 1B) are the most extensively used in consumer products and industrial applications. Despite their inclusion in the Stockholm Convention and global phase-out efforts due to health and environmental concerns, they persist widely in the environment.^35,36^ Continuously released and resistant to effective degradation and removal,^37^ they have become global contaminants, frequently detected in freshwater, surface water, atmospheric aerosols, cloud droplets, and precipitation.^15,16,38–42^ Moreover, PFOA and PFOS have been detected even in remote regions such as the High Arctic.^43^ Ongoing release of these compounds stems from production in regions with fewer regulations, the continued use in applications where no suitable alternatives exist, and their substitution with less-regulated perfluorinated acids, whose environmental effects remain largely unknown.

PFAS and bacterial INs are likely collocated in several critical environmental zones, as they are not only distributed and deposited by similar atmospheric and hydrological pathways^3,8,17,44–47^ but may effectively travel as coupled entities due to the adsorption of PFAS onto bacterial cell surfaces.^48^ Accumulation zones include atmospheric aerosols, clouds, marine systems, and soils.^3,7,11,15,45,49–51^ PFAS can additionally accumulate in plants via root uptake from contaminated soil and translocation within plant tissues^52^ and direct deposition from precipitation or human-generated emission,^47,53^ which is relevant given that bacterial INs are predominantly plant-associated. Moreover, PFAS are potent surfactants that preferentially accumulate at air–water interfaces, such as cloud droplets,^19^ surface water microlayers,^44^ where concentrations can exceed bulk values by several orders of magnitude, as well as in sea spray aerosols that facilitate atmospheric transport of PFAS.^45^ This interfacial enrichment promotes their colocalization with bacterial INs in microscopic aqueous environments, creating conditions of prolonged interaction during atmospheric transport and codistribution, with potential cascading effects on ecosystem health, biodiversity, and climate-relevant freezing processes.

Recent studies have shown that PFOS can be incorporated into lipid membranes due to its surfactant properties, thereby affecting essential membrane properties as well as accumulating in bacterial cultures.^54^ In addition, perfluorinated compounds are capable of binding to proteins and forming hydrogen bonds with amino acid residues.^55^ These findings suggest potential mechanisms by which PFAS could interfere with INpro aggregation and membrane-assisted assembly. While PFAS toxicity to human health is well-established,^56,57^ their effects on biological INs and associated ecological processes remain unexplored.

Here, we investigate the impact of PFOS and PFOA on the bacterial ice nucleation activity. We show that exposure to these perfluorinated acids disrupts INpro aggregates and reduces the freezing efficiency across all bacterial IN classes. By elucidating physicochemical mechanisms underlying the PFAS-induced impairment of bacterial ice nucleation, this work provides new insights into how persistent chemical pollutants may influence biologically driven freezing processes with implications for atmospheric and ecological systems.

## Experimental Section

### Materials

Pure water was obtained from a Millipore Milli-Q Integral 3 water purification system (Merck Chemicals GmbH, Darmstadt, Germany), autoclaved at 121 °C for 15 min, and then filtered through a 0.1 μm bottle-top filtration unit (VWR International GmbH, Darmstadt, Germany). Perfluorooctanoic acid, perfluorooctanesulfonic acid (PFOS 40% (w/w) in water), and MOPS buffer were obtained from Sigma-Aldrich (Darmstadt, Germany). The *P. syringae* CiT7 strain was provided by Steven Lindow from the University of California, Berkeley. *P. syringae* bacteria were grown on King B agar for 3 days at 21 °C before assaying.

### TINA Experiments

Ice nucleation experiments were performed using the high-throughput twin-plate ice nucleation assay (TINA), described in detail elsewhere.^58^ In a typical experiment, a bacterial sample with a concentration of 1 mg/mL in a mixture of water/buffer and perfluorinated organic acids was prepared. This sample was serially diluted 10-fold with a liquid handling station (epMotion ep5073, Eppendorf, Hamburg, Germany). For each dilution, 96 droplets with a volume *V*_droplet_ of 3 μL were placed on two 384-well plates and tested with a continuous cooling rate of 1 °C/min from 0 °C to −30 °C with a temperature uncertainty of ±0.2 °C. The droplet freezing was determined by two infrared cameras (Seek Thermal Compact XR, Seek Thermal Inc., Santa Barbara, CA, USA). The obtained fraction of frozen droplets *f*_ice_(*T*), which describes the number of frozen droplets upon cooling, was used to calculate the cumulative number of ice nucleators *N*_m_(*T*) active at a certain temperature *T* per unit mass *m* using the Vali formula^59^

$$N_{m}\left(T\right)=-\ln\left(1-f_{ice}\left(T\right)\right)\times \frac{V}{V_{droplet}}\times \frac{d}{m}$$

where *m* is the mass of bacteria of the initial suspension, *V* is the volume of the initial suspension, and *d* is the dilution factor relative to the initial suspension. The bacterial mass was estimated from the optical density of the prepared suspension. *N*_m_(*T*) is used to qualitatively assign the recorded freezing behavior to different bacterial IN classes by identifying characteristic increases in *N*_m_(*T*), indicating a high abundance of ice nucleators active in the same narrow temperature range. Experiments were performed multiple times with independent samples, which displayed cumulative freezing profiles and maximum IN concentrations reached as in the literature.^25,27^ Background freezing of pure water occurred at approximately −20 ± 2 °C. For freeze–thaw experiments, the bacterial samples were consecutively cooled down to −30° and thawed at room temperature before each subsequent measurement. The ice nucleation efficiency of a sample is represented by its *T*_50_ value, which corresponds to the temperature at which 50% of the droplets (*f*_ice_ = 0.5) are frozen. *T*_50_ values were determined directly from the experimental data.

## Results

Figure 2 presents the results of droplet freezing experiments conducted on bacterial samples of *P. syringae* in water, with the addition of 0.1 wt % PFOS and 0.1 wt % PFOA. The bacterial samples were serially diluted in 10-fold steps, creating concentrations ranging from approximately 1 mg/mL to 1 ng/mL. The cumulative IN number (*N*_m_) was calculated using Vali’s formula and represents the number of active INs per unit weight above a certain temperature. The freezing spectra of bacterial INs in water show a marked increase in *N*_m_ at −2.4 °C and a smaller increase at −7.5 °C, with a plateau below −9.5 °C. The two increases in *N*_m_ indicate that the IN-activity of the bacterial sample originates from at least two distinct IN classes with different ice nucleation temperatures, while plateaus indicate fewer INs active at these temperatures. Based on the observed freezing activity, we assign these INs to classes A and C, respectively. However, logarithmic scaling can obscure the more gradual increase associated with class B activity. When displayed on a linear scale, the presence of class B INs becomes evident in the cumulative freezing spectrum (see Figure S1), consistent with recent identification through IN subpopulation analysis.^27^

**Figure 2:**
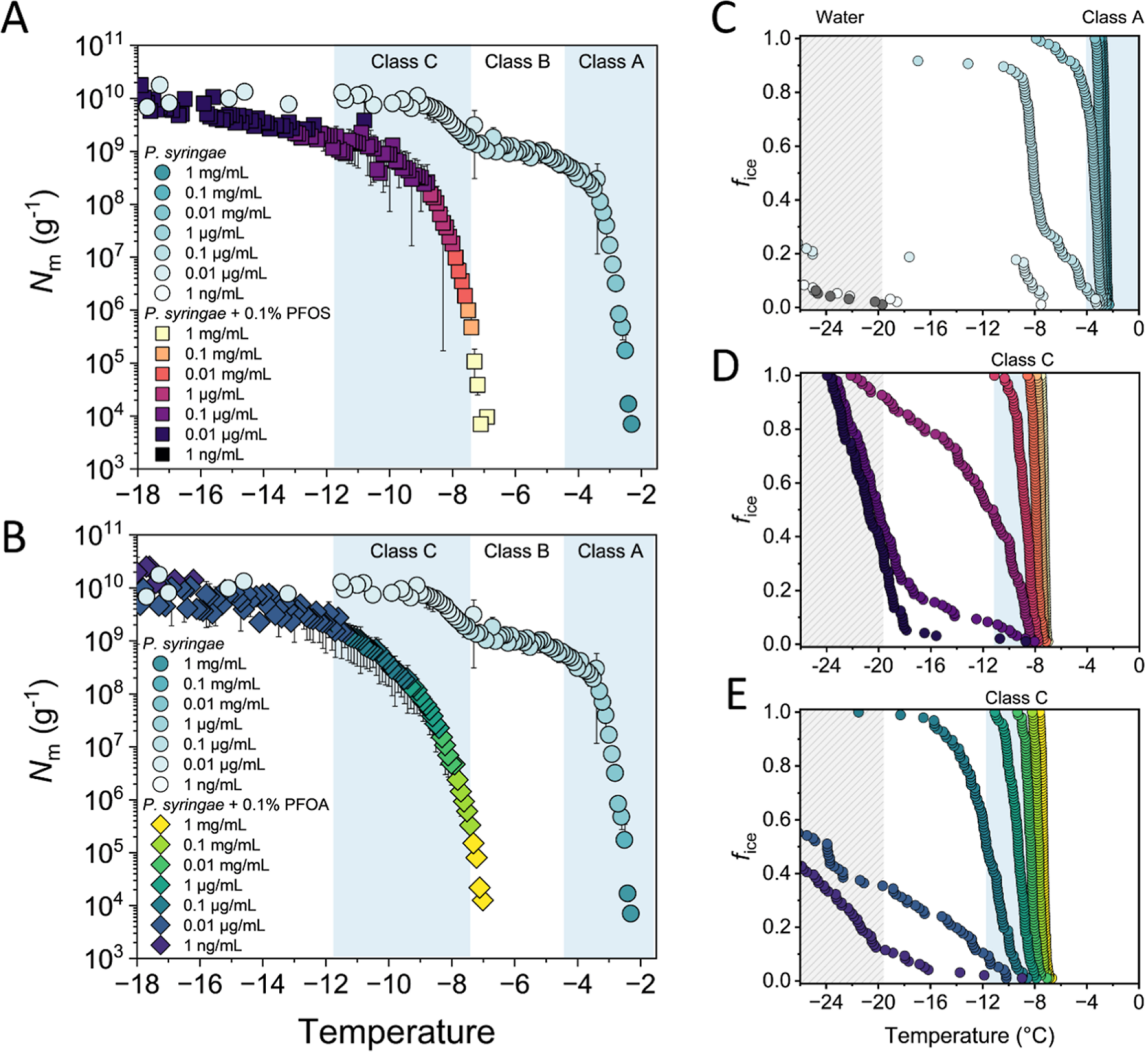
Freezing experiments of aqueous samples containing bacterial INs from *P. syringae* in pure water (light blue circles) and in the presence of 0.1 wt % perfluorinated acids (colored squares and diamonds). For all bacterial samples, concentrations ranging from 1 mg/mL to 1 ng/mL were tested. (A,B) Cumulative number of IN per unit mass of sample (*N*_m_) plotted against temperature for bacterial INs in the presence of PFOS (A) and PFOA (B). (C) Fraction of frozen droplets (*f*_ice_) for different dilutions of *P. syringae* in pure water; (D,E) *f*_ice_ for different aqueous *P. syringae* dilutions in the presence of 0.1 wt % PFOS (D) and PFOA (E). Symbol colors in (C–E) represent different concentrations and are identical to the concentrations shown in (A,B). The blue-shaded regions represent the temperature ranges for class A (*T* > −4.4 °C) and class C (*T* < −7.6 °C). The gray-shaded region indicates when pure water (gray circles in (C)) freezes in our system (below −20 °C). Error bars represent the standard deviation from multiple independent experiments.

Exposure to 0.1 wt % PFOS substantially alters the freezing spectrum of bacterial INs, eliminating their ice nucleation activity at higher subzero temperatures. While the highly efficient class A and class B activity is completely suppressed, only a single rise in *N*_m_ centered at −7.7 °C remains. Treatment with 0.1 wt % PFOA produces similar results, with the remaining activity centered at −8.2 °C. Importantly, the observed freezing shifts cannot be attributed to colligative freezing point depression, as PFOS and PFOA do not act as freely dissolved solutes. Instead, their strong surfactant character promotes preferential accumulation at interfaces, including the water–protein interface, where they can perturbate the structure of INpro assemblies. These findings indicate that both PFOS and PFOA preferentially disrupt large INpro aggregates responsible for class A and B activity while partially preserving the activity of smaller class C aggregates. However, class C INs also exhibit reduced activity, as evidenced by a decrease in the cumulative IN number within the class C range, leading to a gradually rising freezing plateau below −12 °C. Indeed, the initial class C concentration at −7.5 °C is reduced to 0.02% by PFOS and 0.01% by PFOA, confirming that both compounds also impair the integrity of smaller class C aggregates.

Comparison of droplet freezing statistics between bacterial samples in water versus those containing perfluorinated compounds confirms this overall decrease in ice nucleation efficiency. For this, we analyze the fraction of frozen droplets (*f*_ice_), which represents the proportion of droplets frozen at a specific temperature relative to the total sample. Figure 2C shows that most bacterial concentrations in pure water promote freezing temperatures within or near the class A region. In contrast, Figure 2D,E demonstrates that PFOS and PFOA reduce ice nucleation activity to class C INs only. Additionally, less concentrated samples exhibit even lower freezing temperatures, becoming as inactive as water. These results indicate that PFOS and PFOA degrade both larger and smaller INpro aggregates, thereby reducing the overall ice nucleation activity across the full freezing temperature range of bacterial INs. Importantly, the maximal *N*_m_ concentration reached at the plateau of the freezing spectrum, which reflects the total number of INs present, does not change substantially. This is consistent with a stable number of INpros and suggests that PFAS exposure primarily affects aggregate size and assembly rather than protein expression of the bacteria on the time scale of the presented experiments.

Having demonstrated the detrimental effects of PFOS and PFOA on bacterial INs, we next determined the threshold concentration at which both perfluorinated acids affect ice nucleation activity. Figure 3 illustrates the changes in *f*_ice_ of the highest concentrated (1 mg/mL) bacterial solutions of *P. syringae* exposed to increasing PFOS and PFOA concentrations. At 0.001 wt % PFOS, the initial class A activity centered at −2.6 °C slightly shifts to lower temperatures, while a 5-fold increase to 0.005 wt % PFOS abolishes class A activity completely, converting them into the less efficient class C INs. This effect is even more pronounced at the previously tested concentration of 0.1 wt % PFOS. A similar trend is observed for PFOA exposure. Our findings suggest that as little as 0.001 wt % PFOS and 0.005% PFOA, corresponding to a mass concentration of 10 and 50 mg/mL, respectively, is sufficient to induce damage to class A aggregates, which are responsible for the high freezing efficiencies of ice-nucleating bacteria.

**Figure 3:**
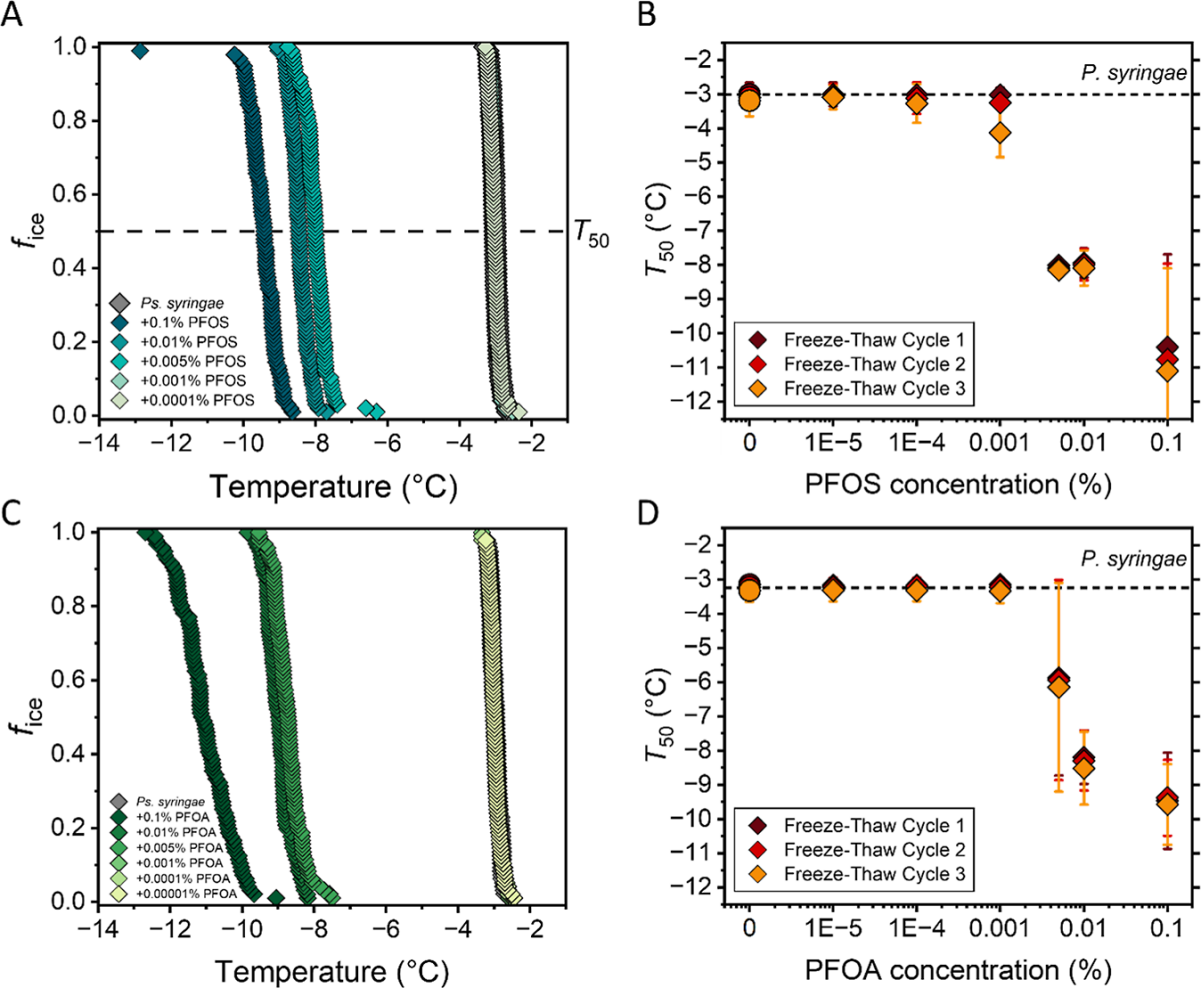
Freezing experiments of aqueous solutions of bacterial INs from *P. syringae* at different PFAS concentrations. (A,C) Fraction of ice (*f*_ice_) for highly concentrated (1 mg/mL) bacterial samples, which exhibit only class A activity in water (gray) with different concentrations of (A) PFOS and (C) PFOA. (B,D) Freeze–thaw experiments of untreated class A INs (circles) and exposed to different amounts of (B) PFOS and (D) PFOA (diamonds). For each freeze–thaw cycle, *T*_50_-values of the bacterial samples as a function of PFOS and PFOA concentration are displayed. Error bars represent the standard deviation from multiple independent experiments.

Additionally, we observe that PFOS-treated bacterial INs exhibit reduced stability toward repetitive freezing, as indicated by shifts in their *T*_50_ values, which represent the temperature at which 50% of droplets are frozen and serve as a measure of ice nucleation efficiency. In Figure 3B, PFOS-exposed samples at the threshold concentration display a *T*_50_ shift from −3.0 °C to −4.1 °C after only three freeze–thaw cycles, whereas untreated controls show no change. This result demonstrates that PFOS further destabilizes INpro aggregates, progressively inactivating them over their bacterial lifetime when they are subjected to multiple freezing events. In contrast, lower PFOA concentrations do not show similar destabilizing effects on bacterial INs. PFOS’s stronger membrane affinity and membrane partitioning,^60,61^ attributed to its sulfonate headgroup and enhanced hydrophobic interactions with lipid bilayers, may underlie this instability by perturbations of the bacterial membrane, which is essential for maintaining INpro aggregate stability.^29^ At higher PFOS levels, class A aggregates fully disintegrate and minor *T*_50_ variations are observed during multiple freeze–thaw cycles for residual activity. We propose that residual class C dimers, which are sensitive to even small mismatches in protein alignment and intramolecular spacing within the dimer structure,^62^ may be more susceptible to misalignments after PFOS exposure and become further destabilized during successive freezing events, leading to their gradual inactivation.

Next, we evaluated the pH changes in PFOS-treated bacterial INs. Figure 4A reveals that the reduction in freezing efficiency, expressed in *T*_50_ values, with increasing PFOS/PFOA concentrations is accompanied by progressively lower pH levels of the bacterial samples. These findings confirm that PFOS-mediated acidification impedes INpro aggregation, consistent with previous studies on the pH sensitivity of bacterial INs,^30^ indicating consequent perturbations of electrostatic interactions relevant for INpro assembly. However, restoring the pH of PFOS-treated samples to control levels only partially recovers the activity, demonstrating that acidification alone does not account for the observed loss of IN activity. In Figure 4B, the addition of 0.1 wt % PFOS to water completely eliminates class A activity, shifting the *T*_50_ to −9.4 °C. Stabilizing the pH using a buffer does not fully counteract the effect, and the *T*_50_ regains only a value of −4.2 °C instead of the initial −2.6 °C. This temperature corresponds to class B activity and aligns well with the freezing temperature predicted for INpro tetramers, whereas freezing around −2.6 °C is associated with 16-mers.^27^ As tetramer formation requires fewer interacting units as the predicted 16-mers for highly efficient class A activity,^27^ these results suggest that PFOS and PFOA alter not only protein–protein interactions but also reduce the probability of highly coordinated multimer assemblies. These additional impairments are likely linked to the pronounced membrane affinity of PFOS and PFOA, which may integrate into the bacterial outer membrane, alter its properties, and thereby hinder functional INpro assembly. The failed restoration of larger multimers suggests a spatial separation of proteins, potentially caused by PFAS-induced increases in membrane fluidity and higher protein mobility.^63,64^ Consequently, smaller, moderately active multimers are more likely to form than larger assemblies, which require multiple closely positioned proteins. Thus, while buffering mitigates acidification effects, it cannot re-establish the close spatial arrangement required for highly coordinated multimer formation, and class A activity is therefore not regained. Together, these findings demonstrate that perfluorinated acids not only are highly effective in destabilizing and disrupting functional INpro aggregates by acidification but also may integrate into the bacterial cell membrane, exerting a multifaceted, long-term effect on bacterial INs.

**Figure 4:**
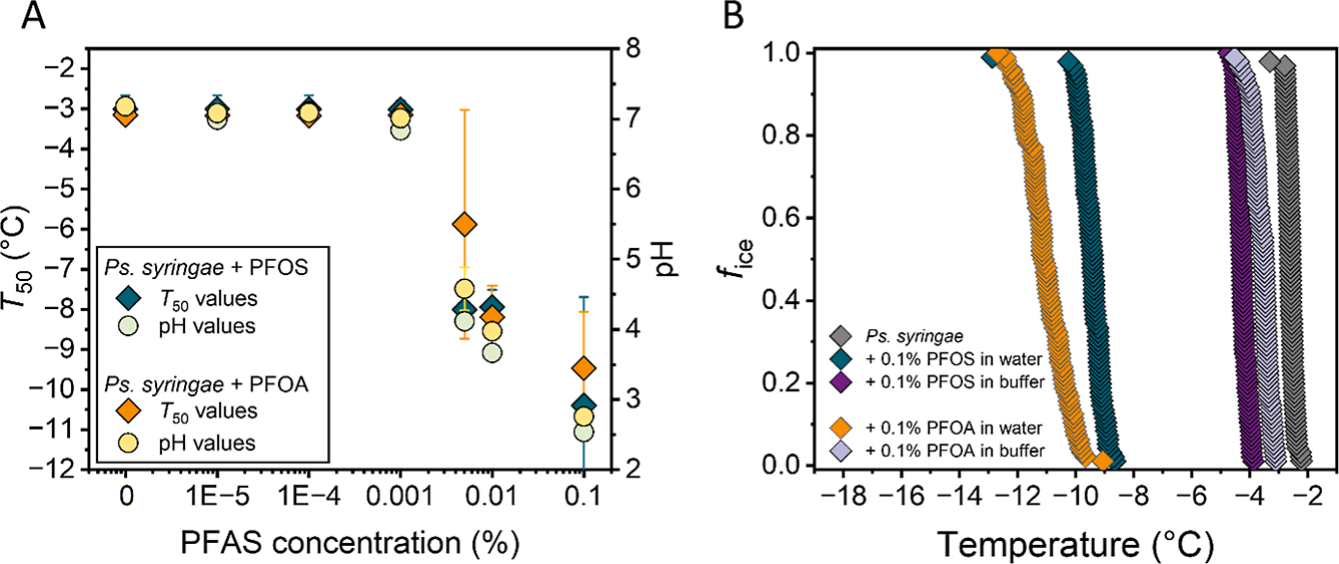
pH measurements and freezing experiments of aqueous solutions of bacterial INs from *P. syringae* at different PFOS and PFOA concentrations in water and in buffered solution. (A) pH values (circles) and *T*_50_ freezing efficiency (diamonds) for PFOS- and PFOA-treated samples in water. Error bars represent the standard deviation from multiple independent experiments. (B) Fraction of ice (*f*_ice_) of untreated samples in water (gray), with 0.1 wt % PFOS in water (green) and in buffered solution (purple) as well as 0.1 wt % PFOA in water (orange) and in buffered solution (light purple).

## Discussion

Our results show that PFOS and PFOA, two prevalent environmental pollutants of the class of perfluorinated organic acids, impair bacterial ice nucleation by disrupting highly efficient INpro aggregates. Because PFOS and PFOA were introduced only after bacterial growth, protein expression, membrane insertion, and aggregate assembly were complete, and on time scales that preclude substantial changes in protein synthesis, the observed loss of activity is most consistent with disruption of pre-existing aggregates rather than downregulated INpro expression. This is further supported by constant IN numbers, indicating that PFAS exposure affects aggregate organization rather than the IN abundance.

The functional assembly of INpros into multimers with high freezing efficiency crucially relies on electrostatic interactions, making these aggregates highly sensitive to pH and ionic perturbations.^27,30,31^ INpros exhibit a distinct charge distribution, with spatially separated negatively charged regions and clusters of positively charged residues, suggesting that multimerization is driven by attractive electrostatic interactions between complementary protein surfaces.^27^ We propose that PFOS and PFOA reduce the INpro activity through a coupled mechanism involving proton-mediated charge screening and direct electrostatic interference. Due to their strong acidity, both compounds introduce excess protons into solutions that can neutralize negatively charged residues, thereby weakening electrostatic interactions critical for multimer stability. In addition, as PFOA is mostly and PFOS is fully deprotonated under the experimental conditions, their anionic headgroups can directly interact with positively charged regions of the protein surface, competing with protein–protein interactions and potentially introducing steric constraints through interfacial accumulation on the protein surface. Together, these effects likely destabilize INpro multimers by disrupting the electrostatic interactions required for the formation and stability of highly efficient ice-nucleating aggregates.

Freezing experiments further reveal that both acids also disintegrate class C INs, which have been attributed to INpro dimers and usually exhibit high stability against external factors.^30,65,66^ However, even subtle shifts in their intramolecular spacing, as small as 0.1 nm, can significantly reduce dimer activity, since the ice nucleation sites of both proteins must align precisely.^62^ The >99.98% decline of the initial cumulative number of class C INs (Figure 2) indicates that perfluorinated acids derange this alignment, possibly through structural protein damage or impairment of the assembly facilitating bacterial membrane, thus resulting in a broad range of lower freezing temperatures and, ultimately, loss of activity. Future studies could explore PFOS-induced disruption of purified class C dimers by monitoring size changes using DLS or intramolecular distance alterations via FRET-based assays, although residual membrane fragments and potential interference of dimerization from fluorophore labeling represent experimental challenges in such approaches.

PFOS additionally destabilizes aggregates at lower concentrations during multiple freezing cycles, an effect not observed in the PFOA-treated samples. This is likely due to PFOS’s stronger membrane affinity and membrane partitioning properties stemming from its sulfonate headgroup and enhanced hydrophobic interactions with lipid bilayers.^60,61^ Furthermore, normalizing the pH in PFOS-treated samples fails to restore initial IN activity. We propose that PFOS not only disrupts electrostatic interactions between INpros but may also integrate into bacterial membranes upon prolonged exposure, as supported by studies showing that PFOS interacts with cell membranes and significantly increases the fluidity of the membrane.^54^ As membrane fluidity is a critical factor for efficient INpro aggregation and the capacity to form class A aggregates is reduced in fluid membranes,^27,67^ this poses an additional factor that may adversely affect bacterial INs in the long term. Future studies may systematically examine changes in membrane fluidity and their effects on outer membrane-embedded INpros via fluorescent membrane probes or single-molecule tracking of labeled INpros to assess their dynamics. Our findings highlight the multifaceted impact of perfluorinated acids on bacterial ice nucleation, with implications for understanding the toxicity of environmental PFAS.

## Conclusions

We have demonstrated that PFOS and PFOA impact the formation of INpro aggregates within the bacterial membrane, diminishing the ice nucleation efficiency of class A, B, and C INs by a coupled mechanism involving charge screening, interruption of electrostatic interactions, and membrane fluidization. Further, we observe that lower PFOS concentrations are sufficient to destabilize INpro aggregates, rendering them vulnerable to repetitive freezing. Our findings are crucial for understanding the impact of PFAS contamination on biological ice nucleators and related freezing processes. Although we detect the observed effects at milligram per liter concentrations, combined exposures of various perfluorinated acids could pose a significant influence on bacterial INs. Furthermore, PFOA and PFOS levels are reported to be increasing due to the ongoing release and the lack of effective removal and degradation methods, with detected concentrations rising from ng/L to μg/L levels.^68^ Future research should focus on investigating the long-term effects of PFAS exposure on bacterial INs in natural environments, considering potential bioaccumulation through membrane partitioning^64,69^ due to surfactant properties of these compounds and combined exposures. This membrane association is concerning because INpro-producing bacteria are globally abundant in atmospheric aerosols and cloud droplets, and their inactivation could systematically alter atmospheric freezing processes, such as mixed-phase cloud glaciation and precipitation efficiency, thereby affecting precipitation patterns and the global hydrological cycle. Additionally, studies exploring the impact of these compounds on other biological ice nucleators and their broader ecological consequences are warranted. This research highlights the importance of ongoing environmental monitoring of PFAS levels and the need to develop effective strategies for PFAS remediation, which is crucial for protecting biologically mediated ice nucleation processes that contribute to atmospheric and hydrological dynamics.^3,4,13,14^

## Supplementary Material



## Notes

Cumulative freezing spectrum of PFOS- and PFOA-treated bacterial samples in water on a linear scale (PDF)
