# Supplementary material for: Impact of Perfluorinated Organic Acids on Bacterial Ice Nucleators
Source: J Phys Chem B. 2026 Feb 10;130(7):2068–76. doi: 10.1021/acs.jpcb.5c07036 (PMC12926935; doi:10.1021/acs.jpcb.5c07036)
Supplement: Supplementary file 1 [file jp5c07036_si_001.pdf]

Supporting Information:

Impact of Perfluorinated Organic Acids on Bacterial Ice Nucleators

Authors: Galit Renzer[1]*, Rosemary J. Eufemio[2], Mischa Bonn[1], and Konrad Meister[1,2]

Affiliations:

[1]Department for Molecular Spectroscopy, Max Planck Institute for Polymer Research, 55128 Mainz, Germany

[2]Department of Chemistry and Biochemistry, Boise State University, 83725 Boise, ID, USA

Correspondence to: renzerg@mpip-mainz.mpg.de

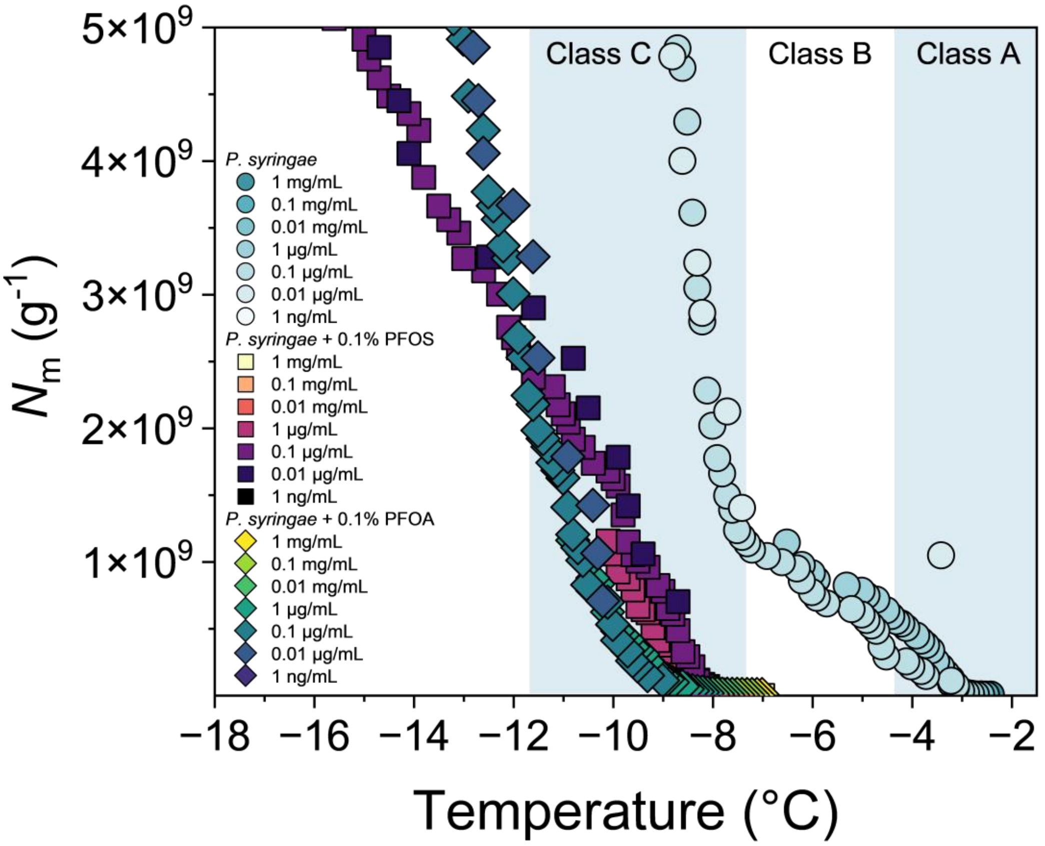

Figure S1: Linear scaled cumulative freezing spectra of aqueous solutions of bacterial INs from *P. syringae* in pure water and in the presence of 0.1 wt% PFOS and 0.1 wt% PFOA. For all bacterial samples, concentrations ranging from 1 mg/mL to 1 ng/mL were tested.
